# Lectures on Practical Surgery

**Published:** 1877-12

**Authors:** 


*Lectures on Practical Surgery.* By H. H. Toland, M. D., Professor of the Principles and Practice of Surgery and Clinical Surgery in the Medical Department of the University of California. With numerous illustrations. Philadelphia: Lindsay & Blakiston, 1877. Buffalo: T. H. Butler. pp. 508, price, cloth $4.50, leather $5.00.

A veteran surgeon who has acquired wealth and fame through the legitimate exercise of his profession, and as a teacher in it, could scarcely fail to have some marvels to relate. It appears that "Tolands' luck in surgery" has, among his co-workers, passed into a proverb. He is too busy about other things to write a book, and his class employed one of the best stenographers of San Francisco to take down his lectures. They were delivered extemporaneously, and the flavor of the book must necessarily differ from one that is written. Removal of an entire os frontis which was necrosed from syphilitic infection, p. 498, and a complete new growth of the same, is among the unusual operations and results mentioned. Compound dislocation of the ankle joint was in one case sufficiently recovered in forty-six days, to allow the patient to attend to his business in a neighboring city.

The author says, "I have been practicing surgery for thirty years, and yet I have neither lost a patient on the table, nor within five days after the operation was performed."

Again, "I have never had, during a long and extensive surgical practice, to remove the dressings but once on account of hemorrhage. He prefers the bilateral operation in lithotomy, and the circular operation in amputations, and never tries to heal a stump by first intention. He says "I would as soon think of committing suicide as of placing an oblique fracture of the tibia in an ordinary fracture box filled with either sand, sawdust, or any of the other substances used for that purpose."

"I have never had a case of simple fracture in which ulceration of the skin resulted either from inflammation, or from the improper pressure of bandages, or splints, except in the first case treated."

The book is elementary in character as is much of the best instruction in Medical Colleges. Dupuytren and Gross are the men whom he most quotes, admires and follows, and they are recognized as leaders in an army of heroes. He does not so much as mention the antiseptic method.

The teachings of thirty years ago are not the teachings of to-day. Perhaps our author might, with justice, be called a little old fashioned, but his success, as we learn it from this volume, has been remarkable. Accuracy in diagnosis and carefulness in prognosis, with keen observation, a brave heart, a cool head, and a steady deftness of hand, with close attention to, and a love of his profession and the best possible preparation for his work, are the apparent elements of his somewhat unusual success. M. B. M.

